# Possible mechanism and clinical potentials of allostery

**DOI:** 10.1186/2001-1326-3-18

**Published:** 2014-07-03

**Authors:** Peixin Huang, Elena López Villar

**Affiliations:** 1 Liver Cancer Institute, Pulmonary Department, Fudan University Zhongshan Hospital, Shanghai 200032, China; 2 Department of Oncohematology of Children, Hospital Universitario Niño Jesús, Avda. Menéndez Pelayo, NO 65, 28009 Madrid, Spain

**Keywords:** Allostery, DNA, Disease, Drug

## Abstract

Allostery is involved in the dynamic regulation of biological functions in proteins. Advances in allostery research have recently drawn great interest and brought allostery closer to the clinic. The present commentary describes the mechanism by which allostery may involve in from a cell-wide view and its contribution to the discovery of new therapeutics to diseases.

## Background

Allostery is a universal phenomenon whereby an effector molecule combining with a (allosteric) site on the protein surface leads to a functional change through alteration of shape and/or dynamics, to regulate protein activity. Effector perturbations can result from a wide range of biological and physical phenomena, including the binding of a small effector molecule, post-translational modifications, protein binding, temperature changes, and pH changes. Allostery takes place in all dynamic proteins, single chains, and in RNA and DNA polymers.

## Main text

Numerous studies demonstrated the importance of the allostery on the protein level, such as MAP kinase, G-protein-coupled receptor (GRCR) kinase, glucokinase (GCK), Hemoglobin S etc. to involve in gene regulation [1-4]. Evolution has exploited allostery as a crucial protein property and optimized it for function [5,6]. Recently, a series of studies reported that the allostery also occurred through RNA and DNA, in addition to the protein level. Three DNA-protein pairs (Cy3B-labled DNA binding domain of glucocorticoid receptor (GRDBD) together with BamHI, Lac repressor (LacR) with EcoRV, or LacR with T7 RNA polymerase) were used to validate and quantitatively characterize the allostery [7-9]. The deformation of the double-helical structure was suggested to be the origin of DNA allostery. However, the altered distance of half a helical turn might affect the evolution with fine-tuning capability of adjusting relative kinetics in regulatory networks. Allostery through DNA was found to be more relative to the placement of regulatory elements such as the transmission of structural influences and switches between canonical structural models.

## Discussion

However, people gradually found that the fundamental importance of allostery is not in the protein or DNA level but, rather, on the cells. From a cell-wide view, allosteric changes can function through a number of pathways which are communicated within, across and between cells. Allostery propagates via molecular cooperativeness and recognition specificity, and affects consecutive components in cellular pathways. The main standpoints to descript how allostery works are thermodynamics, free energy landscape of population shift, and structure [10,11]. Allosteric proteins could activate the conformation of different kinase families and may share a similar structure and the same mechanism. Upstream allosteric sites could switch from inactive αC-helix-out state (OFF) to the active αC-helix-in conformation (ON) [12]. It promotes the inter-connection with several signaling pathways and the formation of allostery network linkage. When mutations cause a high population of ON or OFF states, or lead to an irreversible change in active site shape and dynamics, the allosteric diseases occur [13]. Based on such principle, allosteric drugs targeted at those structures and pathways will provide the possibility of new therapeutics. These pathways may include PI3K/Akt1, MEK1, PDK1 and other protein kinases family pathways [14]. For example, a new model for allosteric regulation of phenylalanine hydroxylase has been studied and ACT interface may be the potential molecule target for therapy [15]. Allosteric ligands bind to protein receptors such as GPCRs also provide the ways for allosteric drugs screening and leading to potential therapeutic benefit [16].

## Conclusion

**Figure 1:**
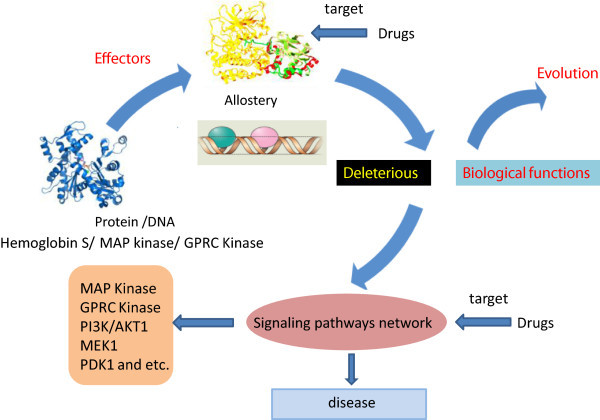
**Allostery in gene regulation.** Effector molecules bind at the protein's allosteric sites, leading to the changes of protein activity. Allostery of protein or DNA then play their respective biological roles through similar signaling pathways in evolution or deleterious physiological process to cause diseases. Targeting signaling pathways or allosteric sites may provide promising therapeutic choices for new drugs.

A collection of recently published papers suggest that allostery of protein or DNA play the respective biological roles in evolution or physiological process. The perturbation, such as mutation, phosphorylation and reaction with small molecules at any site in the protein or DNA structure, leads to a shift in the distribution of the conformational states. The irreversible changes in the structure or the abnormality of switch in allostery are the cause of diseases. Those allosteries may share similar signaling pathways and form network regulation in disease development and may provide promising therapeutic choices for new drugs (Figure 1). Therefore, allostery plays a key role in gene regulation and can be a new alternative to develop new therapies for diseases. Effects of allosteric changes in the pathway should gain more special attentions from a comprehensive and dynamic network perspective.

## Competing interests

The authors declare that they have no competing interests.

## Authors’ contributions

PH, ELV collaborated in order to publish this article and to carry out future research studies. Both authors read and approved the final manuscript.
